# Exposure Type and Duration Determine Ecotoxicological Effects of Cyanobacteria Anatoxins on the Benthic Amphipod *Hyalella azteca*

**DOI:** 10.3390/toxins17110554

**Published:** 2025-11-07

**Authors:** Isabelle Kamalani Yogeshwar, Erwin J. J. Kalis, Juergen Geist, Sebastian Beggel

**Affiliations:** Aquatic Systems Biology Unit, TUM School of Life Sciences, Technical University of Munich, D-85354 Freising, Germany

**Keywords:** anatoxin-a (ATX), homoanatoxin-a (HATX), dihydroanatoxin-a (dhATX), cyanotoxins, toxins, benthic cyanobacteria, *Tychonema*, macroinvertebrates, *Hyalella azteca*

## Abstract

Cyanobacteria can pose a threat to aquatic organisms by their ability to produce toxins such as neurotoxic anatoxins. Although cyanobacteria and their effects on aquatic fauna have been a research focus for a long time, the interactions between benthic cyanobacteria and benthic invertebrates are still largely unknown, especially with regard to how invertebrates cope with cyanotoxins which they are exposed to in their habitat. This study characterizes the effects of anatoxins on the benthic macroinvertebrate *Hyalella azteca*. In a first test, organisms were exposed to synthetically produced anatoxins dissolved in the ambient aqueous phase. In a second test, organisms were exposed to natural anatoxins within intact *Tychonema* cells as their sole food source. Over 10 days of aqueous exposure to anatoxins, survival of *H. azteca* was not affected, even at the highest nominal concentrations of 587.37 µg/L ATX and 590.31 µg/L dhATX. Over 42 days of dietary exposure to natural anatoxins, *H. azteca* readily accepted *Tychonema* as a food source. Survival, growth, reproductive success and storage compound concentrations (glucose, glycogen, lipid and protein) in the organisms’ tissue, all assessed in the same individuals, were reduced. These findings suggest that the ecotoxicological effects of anatoxins on aquatic invertebrates not only depend on their concentration, but even more so on the type and duration of exposure. Furthermore, cyanobacteria like *Tychonema* seem to be insufficient as source of energy if they represent the only available food source.

## 1. Introduction

Cyanobacteria are distributed worldwide [1,2]. Under certain conditions, such as high temperatures and long exposure to sunlight, they can form blooms [2,3,4,5,6]. These blooms can pose a threat to a variety of organisms because some cyanobacteria species are able to produce toxins [2,3,4,6]. These cyanotoxins can be neurotoxins, hepatotoxins, cytotoxins or dermatotoxins and can be produced by both planktic and benthic species [4,5,7,8,9,10]. Depending on their mode of action, they can be lethal within hours or even minutes in a wide diversity of organisms [4]. Cyanotoxins have mostly gained attention due to animal poisonings, but were also found to harm humans [4,5,7,8,9,10,11,12,13,14,15,16,17,18,19,20].

A very potent cyanobacteria neurotoxin which has been found to be responsible for many cases of poisoning is anatoxin-a (ATX) [8,10,20,21,22,23,24]. ATX can be produced by both planktic and benthic species of numerous cyanobacterial genera [2,7,8,22,25,26,27,28,29,30], including *Tychonema sp*. [10,13,31]. These cyanobacteria store ATX intracellularly but may release it into the ambient water when the toxin-containing cells rupture, e.g., due to shear forces or other mechanical disturbances [4,24].

ATX acts by binding to nicotinic acetylcholine receptors of neuromuscular junctions with a higher affinity than acetylcholine itself [7,23,32,33].

At the binding site, it causes a conformational change, which in turn leads to cation influx, resulting in depolarization of the cell and subsequent steps in the signaling cascade which are usually triggered by the binding of acetylcholine. However, since ATX, unlike acetylcholine, cannot be degraded by acetylcholinesterase after binding to the receptor, the cation channel is desensitized, which results in overstimulation of the muscles [23]. A sufficient dose of ATX results in convulsions and death from respiratory paralysis within a few hours or even minutes, which has been reported in mice, rats, fish, ducks and calves [4,7,23,34].

There are several analogs of ATX that are also naturally produced by cyanobacteria [2,4,35], of which homoanatoxin-a (HATX) and dihydroanatoxin-a (dhATX) are the most common [2]. HATX is a methylated variant of ATX, which has a similar toxicity to ATX [4,7,22,36,37]. DhATX appears to be less toxic, although this depends on the test organism and the exposure pathway [22,38,39]. However, no detailed information is available on the comparability of the toxicities of the different anatoxin analogs. In this study, the term anatoxins refers to these three anatoxin variants (ATX, HATX and dhATX).

One cyanobacterial genus that produces several anatoxins, including the three mentioned, is *Tychonema* [2,13,35]. There are several species of *Tychonema*, some of which are planktic and others benthic [2,31,40]. It can be assumed that long-term cohabitation of aquatic invertebrates with cyanobacteria like *Tychonema* has resulted in an adaptation in terms of detoxification abilities, which allow them to survive around or even inside the cyanobacteria colonies [41], but the ecotoxicological effects of anatoxins on co-occurring aquatic species remain poorly understood.

So far, most studies focus on the effects of planktic cyanobacteria on vertebrates [9,39,41,42]. Only limited information is available about the interaction between benthic (anatoxin-producing) cyanobacteria and benthic invertebrates [39,41,42,43]. Such information is particularly important since invertebrates often play a key role in the functioning of aquatic ecosystems, contributing, for example, to the degradation of organic matter [44,45] and providing food to higher trophic levels [41,45,46,47]. Global warming, which favors the spread of such anatoxin-producing cyanobacteria [3,6,31,48,49], calls for a need to further investigate their toxicity to benthic invertebrates, including organism-level effects. One such invertebrate key species is *Hyalella azteca*, an epibenthic freshwater amphipod which feeds on plants and algae and sometimes also cyanobacteria [45,50]. Due to its well-established laboratory culturing and its sensitivity to pollutants, *H. azteca* is a suitable model organism for assessing toxicity in freshwater systems [42,51,52,53]. Therefore, and because of its omnivorous feeding behavior and its ecological relevance, *H. azteca* was chosen as the test organism in this study.

The aim of this study was to provide novel insights into potentially harmful effects of anatoxins produced by benthic cyanobacteria on benthic invertebrates. Therefore, we studied the toxicity of the three anatoxin analogs ATX, HATX and dhATX, combining short-term (acute) and long-term (chronic) exposures and considering different exposure types (aqueous and dietary). In a first test, *H. azteca* were exposed to synthetic ATX or dhATX, which was dissolved in the ambient aqueous medium. In a second test, *H. azteca* were exposed to naturally produced dhATX or HATX by providing them with intact cyanobacteria cells from one of two *Tychonema* strains, containing either of these toxins predominantly, as their sole food source.

We tested the hypotheses that (a) acute aqueous exposure to anatoxins has lethal effects on *H. azteca* and that (b) under the premise that *H. azteca* accepts *Tychonema* material as a food source, long-term exposure to anatoxins via the diet has no lethal effects on *H. azteca* but (c) has sublethal effects on the energy storage of *H. azteca,* resulting in reduced growth, reduced reproduction and reduced storage compound concentrations in their tissues.

## 2. Results

### 2.1. Anatoxin Toxicity in Aqueous Exposure, 10d

ATX and dhATX of synthetic origin in nominal concentrations of up to 587.37 µg/L (ATX) and 590.31 µg/L (dhATX) dissolved in the aqueous phase had no significant effect on the survival of *H. azteca*: After the 10-day exposure, survival in all treatments was between 95 and 100%. The differences in survival at the tested concentrations were not significant for either toxin (for ATX conc. 0.74–235.50 µg/L: *χ*^2^ (7) = 5.03, *p* = 0.6565; for dhATX conc. 0.74–235.50 µg/L: *χ*^2^ (7) = 14.04, *p* = 0.0504); for ATX conc. 587.37 µg/L and dhATX conc. 590.31 µg/L: *χ*^2^ (2) = 2, *p* = 0.3679).

### 2.2. Anatoxin Toxicity in Dietary Exposure, 42d

**Figure 1:**
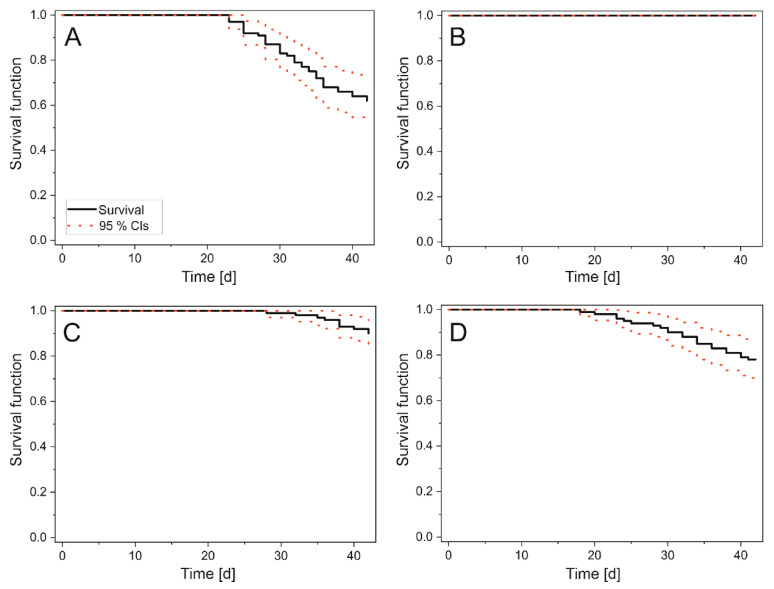
Kaplan–Meier survival estimates for *H. azteca* during a 42-day dietary exposure to anatoxins. Test groups: (**A**) Control 0, not fed. (**B**) Control F, fed with standard crustacean food. (**C**) Tm67, fed with dhATX-containing material from the culture Tm67. (**D**) NC96/3, fed with HATX-containing material from the culture NC96/3. Y-axes show the survival function, with 1 corresponding to 100% survival and 0 corresponding to 100% mortality. X-axes show the time in days. Black lines indicate the survival of the organisms. Step drops in this line indicate that animals have died. Red dotted lines represent upper and lower 95% confidence intervals. N = 5 for (**A**–**D**).

**Figure 2:**
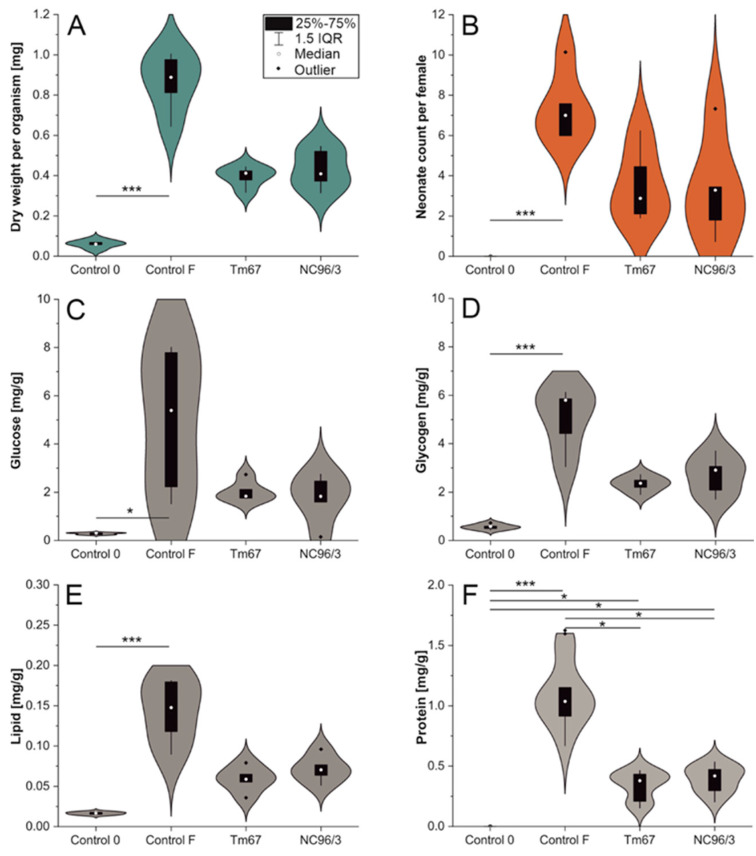
Violin plots of the sublethal effects on *H. azteca* after a 42-day dietary exposure to anatoxins. (**A**) Growth. Y-axis shows the average final dry weight per organism in mg as a measure of growth. (**B**) Reproductive success. Y-axis shows the average number of neonates per surviving female as a measure of reproductive success. (**C**–**F**) Storage compound constellation. Y-axes show the concentration of storage compound per dry weight per organism in mg/g for (**C**) glucose, (**D**) glycogen, (**E**) lipid and (**F**) protein. (**A**–**F**) X-axes show the four test groups: Control 0, not fed. Control F, fed with standard crustacean food. Tm67, fed with dhATX-containing material from the culture Tm67. NC96/3, fed with HATX-containing material from the culture NC96/3. The violin plot illustrates the kernel probability density (i.e., the width of the shaded area represents the proportion of the data located there). White circles represent the median value. Black boxes represent data points between the 25% and the 75% quartiles. Whiskers represent the highest and lowest values inside 1.5 times the interquartile range. Black rhombuses represent outliers. Asterisks denote statistical significance with * = *p* < 0.05 and *** = *p* < 0.001. N = 5 for (**A**–**E**). N = 10 for (**F**).

*H. azteca* readily accepted the anatoxin-containing *Tychonema* material as a food source. After the 42-day exposure, all endpoints were reduced in the *Tychonema*-fed groups compared to the fed control group:

A lethal effect was observed in both *Tychonema*-fed groups. This effect was significantly stronger in the group fed with NC96/3 than in the group fed with Tm67. At the sublethal level, a strong trend was observed for both *Tychonema*-fed groups in terms of reduced growth rates, reduced reproductive success and reduced storage compound concentrations. Table 1 and Figure 1 and Figure 2 show the results for all investigated endpoints. For the corresponding statistics, see Table S1 in the Supplementary Materials.

## 3. Discussion

Toxicity tests with cyanotoxins are essential to better understand the interactions between benthic cyanobacteria and benthic invertebrates and the resulting consequences for aquatic ecosystems. Our study demonstrated that both type (aqueous versus dietary) and duration of exposure substantially determine the ecotoxicological effects of anatoxins on *Hyalella azteca*. Contrary to our initial hypotheses (a) and (b), acute aqueous exposure to anatoxins had no lethal effects on *H. azteca*, whereas long-term dietary exposure did. In line with our third hypothesis (c), this long-term dietary exposure also had sublethal effects on *H. azteca*, including reduced growth, reduced reproductive success and reduced storage compound concentrations in the tissue.

The observed lethal and sublethal effects in the two exposure experiments can be explained by the impact of the anatoxins and their derivatives, as well as by the nutrient composition of the cyanobacterial diet. In particular, the observed absence of mortality (< 5%) during the 10-day aqueous exposure at anatoxin concentrations up to 587.37 µg/L ATX and 590.31 µg/L dhATX is surprising, considering the acute toxicity reported for different aquatic macroinvertebrates in previous studies, even if the reported effect concentrations cover a broad range: 0.49–0.71 µg/L in *Ceriodaphnia dubia* (7d LC_50_, dhATX in diluted cyanobacteria culture water) [42] or 0.79–1.95 µg/L in *Chironomus dilutus* (96h LC_50_, dhATX in diluted cyanobacteria culture water) [42] and up to 2000–14,000 µg/L in *Artemia salina* larvae (24h LC_50_, ATX in lysed and filtered cyanobacterial extracts) [54]. For context, environmental ATX concentrations expressed per L of water samples average between 0.1 and 0.6 µg/L [39,55] and can range from undetectable to higher than 1000 µg/L in some exceptional cases [39,56,57]. In particular, the findings of Anderson et al. [42] indicate a higher sensitivity of *Hyalella azteca* to anatoxins: In their study, the authors exposed *H. azteca* to diluted anatoxin-containing BG11 culture medium for 96 h and found mortality with an LC_25_ of 1.26–5.30 µg/L (strain-dependent), which they suggest was caused by the dominant anatoxin analog dhATX in their test solutions [42]. This discrepancy between the results of Anderson et al. and our results could, on the one hand, be due to species- or clade-specific sensitivity differences. Since *Hyalella azteca* is a species complex and the exact clade is not known for either study, it is possible that different clades with different sensitivities were used. On the other hand, the discrepancy could be due to the different water matrices that were used, since Anderson et al. used diluted anatoxin-containing BG11 medium as their test solution [42], and we used chemical-grade ATX or dhATX in SAM-5S medium. As mentioned in their study, it cannot be ruled out that this BG11 medium also contained other compounds which were not analyzed but could have had a toxic effect [42]. It is therefore possible that their observed effects were not exclusively attributable to the measured anatoxin concentrations. However, due to the different water matrices used and the differing levels of purity of the toxins used, the results of Anderson et al.’s study and our study lack direct comparability.

Although the *Tychonema*-fed groups subject to 42-day dietary exposure had *ad libitum* access to their food source, the anatoxin doses of the ingested *Tychonema* cells were substantially lower than the concentrations of purified anatoxins in the 10-day aqueous exposure experiment. Considering the absence of mortality in the 10-day aqueous exposure in contrast to the observed mortality and sublethal effects in the 42-day dietary exposure, this indicates that the anatoxin concentration alone cannot explain these effect differences. However, an additional nutrient deficiency can. In our experiment with aqueous anatoxin exposure, the test organisms were provided with unlimited energy by *ad libitum* feeding with standard crustacean food. However, in the experiment with dietary anatoxin exposure, the *Tychonema*-fed groups could only obtain their energy from these cyanobacterial cells. Some previous studies report that cyanobacteria contain valuable nutrients and macromolecules which are necessary for the survival, growth and maturation of macroinvertebrates [41,58,59,60,61,62]. However, measurements of glucose, glycogen, lipid and protein concentrations in the tissue of our *Tychonema*-fed test organisms compared to those of the fed Control F group show clearly that the nutritional quality offered by the cyanobacterial cells was much lower than that offered by the control diet with standard crustacean food. This indicates that malnutrition itself is likely to be an important co-driver of the observed mortality and sublethal effects in the *Tychonema*-fed groups. Consequently, exposures to pure strains of cyanobacteria which are atypical in natural environments should ideally be complemented by more realistic exposures to multiple cyanobacteria species as potential food sources for realistic assessments.

Additionally, the lack of energy might have negatively affected potential detoxifi-cation processes. It is likely that *H. azteca*, during their evolutionary cohabitation with cyanobacteria, acquired detoxification abilities to survive in the presence of cyanotoxins or to even use cyanobacteria as a source of nutrition [50]. Detailed information on the mechanisms of such detoxification abilities in *H. azteca* when exposed to anatoxins has not yet been described in the literature. However, previous studies report an alteration in relevant biochemical markers induced by ATX in other organisms, e.g., *Oncorhynchus mykiss* [63]. The upregulated markers include important enzyme activities, such as acetylcholinesterase (involved in neurotransmission), lactate dehydrogenase (involved in energy metabolism), glutathione S-transferase (involved in biotransformation in the liver) and others [63]. The authors suggest that *O. mykiss* may have experienced increased metabolic demands to meet the energetic requirements caused by anatoxin detoxification mechanisms [63]. It is conceivable that *H. azteca* in our study also had increased energy requirements due to detoxification mechanisms triggered by anatoxin exposure in diet, which could not be met by the low nutritional quality of the *Tychonema* cultures as the sole food source. Future studies can investigate this in *H. azteca* and other benthic invertebrates to elucidate the mechanistic background of possible detoxification processes.

The assumption that the mortality observed in the 42-day dietary exposure is due to the anatoxins in addition to nutrient deficiency is supported by the strain-dependent mortality differences in the *Tychonema*-fed groups: Mortality was higher in the NC96/3-fed group, which contained a higher toxin concentration (HATX conc. of 207 µg/g fresh weight), than in the Tm67-fed group (dhATX conc. of 161 µg/g fresh weight). Furthermore, other studies also mention HATX as a more potent neurotoxin compared to dhATX due to its higher binding affinity to the acetylcholine receptor [4,7,22,36,37,38,39].

A slight distortion of the observed effects on growth and reproductive success has to be taken into account due to differing sex ratios across the test groups. *H. azteca* is sexually dimorphic, with males usually being slightly larger than females [64,65], so the sex ratio may affect the final dry weight per organism (measure of growth). Furthermore, males guard females longer during mating and may exhibit competitive behavior when females are limited [64,66,67]. Thus, the sex ratio may also affect reproductive success. In the four groups of our 42-day dietary exposure test, the female-to-male (F:M) ratio of the surviving test organisms, assessed after the end of exposure, averaged 1F:1.7M for Control F, 1F:1.2M for Tm67 and 1.8F:1M for NC96/3 and was not evaluable for Control 0 due to the absence of sexual maturation. Given these sex ratios, without any treatment such as exposure to toxins or nutrient deficiency, the highest reproductive success would be expected in the group with the most females and the least males (NC96/3), and the lowest reproductive success would be expected in the group with the least females and the most males (Control F). In light of this expectation based on the sex ratios, the results of our dietary anatoxin exposure indicate that growth and reproductive success in NC96/3 were even more reduced relative to Control F.

In addition to the anatoxins and the nutrient composition, the exposure scenario seems to be a key factor contributing to the observed ecotoxicological effects: The type of exposure (aqueous versus dietary) and the exposure duration are particularly important in this regard. In general, it is not surprising that aqueous or cell-free exposure and dietary or within-cell exposure lead to different effects. For instance, Werner et al. [68] detected different ecotoxicological effects of aqueous versus dietary exposure to a pyrethroid pesticide on medaka fish (*Oryzias latipes*): They found mortality only in test organisms in aqueous exposure, but no mortality in test organisms in dietary exposure [68]. Different effects depending on aqueous or dietary exposure have also been observed for cyanotoxins [41]: Ubero-Pascal and Aboal [41] report a tendency in previous studies that toxic effects are generally more lethal and severe in cell-free exposure than when cyanotoxins are ingested via diet [41]. This heterogeneity of effects is likely related to the class of cyanotoxins (neurotoxins, hepatotoxins, cytotoxins, dermatotoxins) and the corresponding mechanism of action. However, even toxins within the same cyanotoxin class, in this case neurotoxins, can differ mechanistically. Heterogeneous effects can also occur depending on how the toxin enters the organism [41]. The entry of anatoxins into invertebrate organisms via the skin or gills during aqueous exposure might cause different effects compared to the entry of anatoxins via food intake, where metabolization by digestive enzymes may possibly reduce or increase the toxicity. Furthermore, whether the organisms ingest the cyanotoxins in the purified form or as cyanobacterial extract might play a role. Toporowska et al. [69], who investigated the toxicity of anatoxins and microcystins on *Chironomus spp*., observed lower toxicity for both cyanotoxins when the test organisms were exposed to the pure toxin compared to cyanobacterial extracts containing the toxin, even though the toxin concentrations in the cyanobacterial extracts were approximately 10 times lower than those of the chemically pure standards. Similar effects were observed by Lahti et al. [54], who exposed *Artemia salina* larvae to ATX-containing cyanobacterial extracts of two levels of purity: In the group exposed to lysed and filtered extracts, they found mortality, whereas in the group exposed to extracts that had additionally undergone solid phase fractionation and contained the same amount of ATX, no mortality was observed [54]. Our study shows a similar outcome to those of Toporowska et al. [69] and Lahti et al. [54]. Therefore, in addition to the known cyanotoxins, cyanobacteria or their extracts probably contain other components influencing toxicity that have not yet been characterized.

Furthermore, it is generally not surprising that the duration of exposure has a significant influence on the ecotoxicological effects. Chronic exposure can lead to different effects than acute exposure, e.g., because some cyanotoxins can accumulate in the organism during long-term exposure, where they cause further damage. The accumulation of several cyanobacteria species has already been studied, with most published data focusing on hepatotoxic microcystins [70,71]. In contrast, less data is available on the accumulation of neurotoxins, especially anatoxins, in invertebrates [70], and these are controversial: While some studies suggest that anatoxins can accumulate in invertebrates, such as *Chironomus spp*. [69] or carp (*Cyprinus carpio* L.) [72], Colas et al. [73] report rapid elimination of ATX in medaka fish (*Oryzias latipes*). More research is needed on this topic and, in the case of anatoxin accumulation, on the possible transfer of these anatoxins to other trophic levels in the food chain of freshwater ecosystems. This research could, for example, simulate a simplified food chain in which a lower-trophic species is constantly exposed to anatoxins and then serves as a food source for a higher-trophic species.

We propose a more systematic and harmonized research approach for assessing the toxicity of cyanobacteria and their toxins on aquatic invertebrates in order to improve the comparability of future findings in this field. Although many studies have already been published on the effects of cyanotoxins on aquatic fauna, the findings are often difficult to classify and compare because, for example, different water matrices are used, the toxins are tested in combination with other components or other variables render the findings unusable for direct comparison. For future research, it would therefore be important to define (a) the type of exposure: In addition to cyanotoxins in cyanobacterial extracts, regardless of whether they are cell-free or within cells, it would be advantageous to test a chemically pure standard of the respective cyanotoxin. This would help to clarify the fundamental question of which effects are actually due to the toxins and which are more likely the result of several combined components, some of which may be known to be non-toxic on their own or have so far unknown bioactive properties. Furthermore, if possible, (b) standardized media that mimic the natural habitat water chemistry or provide ideal conditions for the test organisms should be used for test solutions, rather than culture media for cyanobacteria, to avoid unintended synergistic effects. For (c) the selection of test organisms, it would be useful to consider organisms from standing waters as well as from flowing waters, e.g., *Gammarus sp*., which also play an important role in freshwater ecosystems and may exhibit different sensitivities.

Finally, the findings of our study reveal important implications for risk assessment, as tests that rely exclusively on aqueous toxin exposure may overlook or underestimate effects. Based on the mortality observed in our study during dietary exposure, we recommend including dietary exposure in future standard testing frameworks. Such studies could furthermore benefit from measuring feeding rates and using food choice scenarios with more than one cyanobacteria species as a potential food source in order to approximate more natural food preferences of the test organisms. Further research studying the underlying mechanisms at the molecular level is needed to better understand the effects of anatoxins observed on the whole-organism level and potential detoxification processes.

## 4. Conclusions

In this study, we demonstrated that 10-day aqueous exposure to anatoxins has no lethal effects on *Hyalella azteca*, whereas 42-day exposure to anatoxins via pure *Tychonema* diets has both lethal and sublethal effects on *H. azteca*, as indicated by reduced survival, reduced growth, reduced reproductive success and reduced concentrations of glucose, glycogen, lipid and protein in their tissue.

From these findings we conclude that the ecotoxicological effects of anatoxins on aquatic invertebrates like *H. azteca* not only depend on their concentration but more importantly on the type and duration of exposure. Furthermore, cyanobacteria like *Tychonema* seem to be an insufficient source of energy if they represent the only available food source.

For future research, our findings suggest that dietary exposure should be included in future standard testing frameworks for risk assessments. Furthermore, studies focusing on the effects on anatoxins at the molecular level will lead to a better understanding of the mechanistic pathways involved in the stress response. To facilitate the comparability between future research findings on the interaction of benthic cyanobacteria with benthic invertebrates, we propose a more systematic approach with well selected test substances, media and organisms, as well as harmonized endpoints.

## 5. Materials and Methods

### 5.1. Anatoxins

Synthetic forms of ATX and dhATX were purchased as fumarates (ATX: Santa Cruz Biotechnology Inc., Dallas, TX, USA; dhATX: Hello Bio Ltd., Bristol, UK). These were stored at −20 °C until use and only thawed immediately before preparing the stock solutions for the aqueous exposure experiment.

Natural forms of dhATX and HATX within intact cells from two different *Tychonema* cultures were used for the dietary exposure experiment. Toxin contents were determined by the German Federal Environment Agency using LC-MS analysis, as described in a previous study [13]. DhATX was the dominant toxin of the *Tychonema* culture Tm67 (161 µg/g fresh weight), sampled from Lake Mandicho near Augsburg, Germany (coordinates: 48.25869197973319, 10.938291979226513) and subsequently cultured at the Aquatic Systems Biology Unit at the Technical University of Munich (TUM), Germany. HATX was the dominant toxin of the *Tychonema* culture NC96/3 (207 µg/g fresh weight), purchased from the company NORCCA (Oslo, Norway) and subsequently also cultured at the Aquatic Systems Biology Unit at TUM. In this study, these cultures are referred to as Tm67 and NC96/3. In the laboratory, both cultures were maintained in 1 L Erlenmeyer flasks with 1% agar and 600 mL Z8 medium, prepared based on Rippka [74], at 17 °C in a 16:8 h light:dark regime.

### 5.2. Test Organisms

*Hyalella azteca* cultures were obtained from an online aquarist supplier (interaquaristik.de Shop, Biedenkopf-Breidenstein, Germany) and acclimated to laboratory conditions prior to the experiments according to Novak and Taylor [75]. *H. azteca* is a species complex, and their exact clade is not known for this study. The cultures were maintained according to Novak and Taylor [75] in SAM-5S reconstituted water in 5 L beakers equipped each with a 10 × 10 cm gauze square. The cultures were constantly aerated and maintained at 22 °C and a 16:8 h light:dark regime. The culture medium was exchanged weekly and animals were fed with crustacean food flakes (Crusta Menu, Tetra GmbH, Melle, Germany) *ad libitum* unless stated otherwise in the specific descriptions below.

### 5.3. Toxicity Tests

The tests were conducted following the standard procedure described in Novak and Taylor [75]. *H. azteca* individuals aged 7–9 days were extracted from the maintenance culture and then individually transferred to the test vessels using a plastic dropper. Toxicity tests were conducted under static conditions with continuous aeration.

#### 5.3.1. Toxicity Test with Exposure to Dissolved Synthetic Anatoxins

Two stock solutions were prepared by dissolving ATX fumarate or dhATX fumarate in SAM-5S medium. Test solutions were prepared by diluting these stock solutions with SAM-5S medium. The following nominal concentrations were tested for ATX and dhATX: 0 (control), 0.74, 1.47, 2.94, 5.89, 11.78, 23.55 and 235.50 µg/L. Additionally, the highest ATX concentration of 587.37 µg/L and the highest dhATX concentration of 590.31 µg/L were tested. Toxin contents were determined at random by the German Federal Environment Agency after the experiments using LC-MS analysis [13]. However, since the toxin analysis was carried out two months after the end of exposure and anatoxins degrade in water over time, the measured values did not reflect the actual concentrations in the test solutions during the exposure period. To avoid overestimation of effects, we therefore rely on the nominal concentrations for this experiment. The test duration was 10 days, during which survival was measured daily by visual inspection, according to Novak and Taylor [75]. Table 2 gives an overview of the volume of the test solution in the test vessels, the number of replicates and the number of test organisms per test vessel.

#### 5.3.2. Toxicity Test with Exposure to Natural Anatoxins via a *Tychonema* Diet

In a second exposure test, test organisms were offered anatoxins via their diet by providing them with intact cells of the *Tychonema* cultures Tm67 or NC96/3 as the sole food source. For this, some cyanobacteria material was taken from the respective culture with sterile tweezers and directly transferred to the test vessels. These cyanobacteria cells contained the anatoxins intracellularly. The test contained four groups: A first group (= Tm67) was fed with cells from Tm67. A second group (= NC96/3) was fed with cells from NC96/3. One control group (= Control F) was fed with standard crustacean food flakes, and another control group (= Control 0) received no food at all. For each of the four groups, 5 replicates were used, each containing 900 mL SAM-5S medium and 20 test organisms (total n = 400). The three food sources were provided *ad libitum*. Leftover pieces of *Tychonema* material were replaced by fresh material at least three times a week. No feeding rates were measured. During 42 days of exposure, survival was measured three times a week on non-consecutive days. After the exposure period, growth, reproduction and concentrations of storage compounds were measured as other toxicological endpoints. Survival was again determined by visual inspection, see [75]. Growth was assessed by the average final dry weight per organism, and reproductive success was assessed by the average number of offspring per surviving female, according to Novak and Taylor [75]. Storage compound concentrations were assessed by mg storage compound per g dry weight per organism, as detailed below.

#### 5.3.3. Assessment of Storage Compounds

In this study, the term “storage compounds” refers to glucose, glycogen, lipid and protein in the tissue of the test organisms.

For the storage compound measurements, test organisms of the same replicate of a test group were pooled and weighed after 42-day dietary exposure. They were then homogenized, first dry and then in methanol, until a homogenate with a total of 900 µL methanol was obtained. This was performed for each replicate of each test group. Each of these homogenates was then divided into three parts - one part for each of the following three assays.

Glucose and glycogen concentrations in the test organisms were determined photometrically at 625 nm using an anthrone reagent and a sodium sulfate solution, according to the methods established by van Handel [76] and Götz et al. [77]. For the calibration, a 1 mg/mL glucose analytical standard solution was diluted to a final content of 10, 30, 50, 70 and 100 µg glucose in the calibration solutions. Assay precision and repeatability were satisfactory (R^2^ = 0.996, intra-assay CV = 0.08).

Lipid concentrations in the test organisms were determined photometrically at 525 nm using a vanillin-phosphorus reagent, according to the methods established by van Handel [78] and Götz et al. [77]. For the calibration, commercial olive oil was used in a 1:2 methanol:chloroform (v/v) solution, which was diluted to a final content of 25, 37, 50, 75, 100 and 150 µg lipid in the calibration solutions. Assay precision and repeatability were satisfactory (R^2^ = 0.988, intra-assay CV = 0.03).

Protein concentrations in the test organisms were determined photometrically at 595 nm using a Coomassie reagent, according to the methods established by Bradford [79] and Walker [80]. For the calibration, a 1 mg/mL bovine γ-globulin analytical standard solution was diluted to a final content of 1, 2, 5, 10, 20 and 50 µg protein in the calibration solutions. Assay precision and repeatability were satisfactory (R^2^ = 0.992, intra-assay CV = 0.25 due to high variability at the lowest concentration of the test standard).

For the photometric measurements, a UV/VIS spectro-photometer (UVIKON 923, Goebel GmbH, Au in der Hallertau, Germany) was used together with the associated program (UVS900 Lite, version V2.22.6, DuSoTec GmbH, Tiefenbach, Germany). The storage compound concentrations in the tissue samples were calculated using the photometric absorbance, the measured dry weight of the test organisms and the calibration curves.

### 5.4. Statistical Analysis

To determine effects of anatoxin exposure via the aqueous phase as well as via diet, we calculated survival estimates using the Kaplan–Meier procedure. Log-rank tests were used to test the differences between the survival curves of the treatment groups. To test differences between the treatment groups for the sublethal endpoints, non-parametric Kruskal–Wallis tests were used. If test results showed significance, subsequent *post hoc* tests (Dunn–Bonferroni) were performed for pairwise comparisons. Significance was accepted at *p* < 0.05. All analyses were conducted using OriginPro, Version 2024 (OriginLab Corporation, Northampton, MA, USA).

## Figures and Tables

**Table 1:** Results of the 42-day dietary exposure to natural anatoxins within *Tychonema* cells. Survival is indicated by percentage. Growth is expressed as average final dry weight per organism. Reproductive success is expressed as average number of neonates per surviving female (N_n_). Storage compound concentrations are expressed as mg storage compound per g dry weight per organism. SD = standard deviation. n.d. = not detectable. ^a,b^ denote significance between the equally marked groups.

42d Dietary Exposure							
	Survival [%]	Growth [mg ± SD]	Reproduction [N_n_ ± SD]	Storage Compound Concentration [mg/g ± SD]			
				Glucose	Glycogen	Lipid	Protein
Control 0	62 ^a^	0.06 ± 0.02 ^a^	0 ^a^	0.28 ± 0.04 ^a^	0.56 ± 0.11 ^a^	0.02 ± 0.002 ^a^	0 (n.d.) ^a,b^
Control F	100 ^a^	0.87 ± 0.15 ^a^	7.3 ± 1.7 ^a^	4.99 ± 3.04 ^a^	5.05 ± 1.31 ^a^	0.14 ± 0.04 ^a^	1.09 ± 0.27 ^a,b^
Tm67	90 ^a^	0.39 ± 0.05	3.5 ± 1.8	2.03 ± 0.42	2.34 ± 0.32	0.06 ± 0.02	0.34 ± 0.11 ^a^
NC96/3	78 ^a^	0.43 ± 0.10	3.3 ± 2.5	1.76 ± 1.02	2.69 ± 0.80	0.07 ± 0.02	0.40 ± 0.11 ^b^

**Table 2:** Test setup details for the ATX and dhATX aqueous exposure tests (total n = 170).

Tested Toxin	Nominal Concentration [µg/L]	Volume of Test Solution [mL]	Number of Replicates	Number of Organisms Per Test Vessel
ATX	0–23.55	275	6	10
ATX	235.50	275	3	10
ATX	587.37	125	4	5
dhATX	0–235.50	275	4	10
dhATX	590.31	125	4	5

## Data Availability

The raw data supporting the conclusions of this article will be made available by the authors on request.

## Supplementary Materials

The following supporting information can be downloaded at https://www.mdpi.com/article/10.3390/toxins17110554/s1: Table S1: Summary test statistics for the 42-day dietary exposure experiment. Main effects were tested using χ^2^-statistics for non-parametric data. Pairwise comparison for survival data were conducted using log-rank tests (χ^2^-statistics), pairwise comparison for sublethal and biochemical parameters were conducted using Dunn-Bonferroni tests (Z-statistics). For effect size calculation of χ^2^ tests Cohen’s η^2^ was used, for Dunn-Bonferroni tests the rank-biserial correlation r was used. *p*-values showing significant differences are indicated in bold.
