# Supplementary material for: Exposure Type and Duration Determine Ecotoxicological Effects of Cyanobacteria Anatoxins on the Benthic Amphipod Hyalella azteca
Source: Toxins (Basel). 2025 Nov 7;17(11):554. doi: 10.3390/toxins17110554 (PMC12655909; doi:10.3390/toxins17110554)
Supplement: Supplementary file 1 [file toxins-17-00554-s001.zip › toxins-3923270-supplementary.pdf]

Supplementary Materials: Exposure Type and Duration Determine Ecotoxicological Effects of Cyanobacteria Anatoxins on the Benthic Amphipod *Hyalella azteca*

Isabelle Kamalani Yogeshwar, Erwin J. J. Kalis, Juergen Geist and Sebastian Beggel

Table S1. Summary test statistics for the 42-day dietary exposure experiment. Main effects were tested using χ2-statistics for non-parametric data. Pairwise comparison for survival data were conducted using log-rank tests (χ2-statistics), pairwise comparison for sublethal and biochemical parameters were conducted using Dunn-Bonferroni tests (Z-statistics). For effect size calculation of χ2 tests Cohen's η2 was used, for Dunn-Bonferroni tests the rank-biserial correlation r was used. P-values showing significant differences are indicated in bold.

Main effect	Parameter	Number of groups	Total sample size (all groups combined)	Chi square	Df	P-value	Effect size
		k	n	χ^2			Cohen's η^2
	Survival	4	400	58.85417	3	**<0.0001**	0.14
	Growth	4	20	16.07429	3	**0.00109**	0.82
	Reproduction	4	20	14.38136	3	**0.00243**	0.71
	Glucose	4	20	10.07429	3	**0.01795**	0.44
	Glycogen	4	20	15.48	3	**0.00145**	0.78
	Lipid	4	20	15.93714	3	**0.00117**	0.81
	Protein	4	40	33.6957	3	**2.30E-07**	0.85
**Pairwise comparisons**							
	Survival						
	Control 0 vs. Control F	2	200	47.17499	1	**<0.0001**	0.23
	Control 0 vs. Tm67	2	200	23.23906	1	**<0.0001**	0.11
	Control 0 vs. NC96/3	2	200	5.95207	1	**0.0147**	0.03
	Control F vs. Tm67	2	200	10.4767	1	**0.00121**	0.05
	Control F vs. NC96/3	2	200	24.66175	1	**<0.0001**	0.12
	Tm67 vs. NC96/3	2	200	5.86914	1	**0.01541**	0.02

Parameter	Number of groups	Total sample size (all groups combined)	Z-value	Df	P-value	Effect size
	k	n	Z			Rank-biserial correlation r
Growth						
Control 0 vs. Control F	2	10	-4.00892	1	**3.66E-04**	-1.27

Control 0 vs. Tm67	2	10	-1.97773	1	0.28775	-0.63
Control 0 vs. NC96/3	2	10	-2.03119	1	0.25342	-0.64
Control F vs. Tm67	2	10	2.03119	1	0.25342	0.64
Control F vs. NC96/3	2	10	1.97773	1	0.28775	0.63
Tm67 vs. NC96/3	2	10	-0.05345	1	1	-0.02
Reproduction						
Control 0 vs. Control F	2	10	-3.77155	1	**9.73E-04**	-1.19
Control 0 vs. Tm67	2	10	-2.20905	1	0.16303	-0.70
Control 0 vs. NC96/3	2	10	-2.10129	1	0.21369	-0.66
Control F vs. Tm67	2	10	1.5625	1	0.70902	0.49
Control F vs. NC96/3	2	10	1.67026	1	0.56921	0.53
Tm67 vs. NC96/3	2	10	0.10776	1	1	0.03
Glucose						
Control 0 vs. Control F	2	10	-3.10023	1	**0.0116**	-0.98
Control 0 vs. Tm67	2	10	-2.13809	1	0.19506	-0.68
Control 0 vs. NC96/3	2	10	-1.71047	1	0.52307	-0.54
Control F vs. Tm67	2	10	0.96214	1	1	0.30
Control F vs. NC96/3	2	10	1.38976	1	0.98761	0.44
Tm67 vs. NC96/3	2	10	0.42762	1	1	0.14
Glycogen						
Control 0 vs. Control F	2	10	-3.90201	1	**5.72E-04**	-1.23
Control 0 vs. Tm67	2	10	-1.81738	1	0.41496	-0.57
Control 0 vs. NC96/3	2	10	-2.29845	1	0.12922	-0.73
Control F vs. Tm67	2	10	2.08464	1	0.22261	0.66
Control F vs. NC96/3	2	10	1.60357	1	0.65286	0.51
Tm67 vs. NC96/3	2	10	-0.48107	1	1	-0.15
Lipid						
Control 0 vs. Control F	2	10	-3.95547	1	**4.58E-04**	-1.25
Control 0 vs. Tm67	2	10	-1.76392	1	0.46647	-0.56
Control 0 vs. NC96/3	2	10	-2.29845	1	0.12922	-0.73
Control F vs. Tm67	2	10	2.19154	1	0.17048	0.69
Control F vs. NC96/3	2	10	1.65702	1	0.58509	0.52
Tm67 vs. NC96/3	2	10	-0.53452	1	1	-0.17
Protein						
Control 0 vs. Control F	2	10	-5.78312	1	**<0.0001**	-1.83
Control 0 vs. Tm67	2	10	-2.64096	1	**0.0496**	-0.84
Control 0 vs. NC96/3	2	10	-3.14216	1	**0.01006**	-0.99
Control F vs. Tm67	2	10	3.14216	1	**0.01006**	0.99
Control F vs. NC96/3	2	10	2.64096	1	**0.0496**	0.84
Tm67 vs. NC96/3	2	10	-0.5012	1	1	-0.16
